# Extracellular vesicles from miR-146a-overexpressing mesenchymal stem cells reduce ligature-induced periodontitis by modulating inflammatory cytokines

**DOI:** 10.1186/s12865-026-00839-3

**Published:** 2026-05-09

**Authors:** Wen Chu, Yinuo Zhang, Shiying Shen, Zhilu Wang, Yanxu Guo, Yanfang Yu, Dahai Huang, Nara Davtyan

**Affiliations:** 1 Department of Oral Preventive Health Care, The Affiliated Hospital of Yunnan University, Kunming, 650021 China; 2 Department of Prosthodontics, The Affiliated Hospital of Yunnan University, Kunming, 650021 China; 3 Yerevan State University, Yerevan, Armenia

**Keywords:** Periodontitis, Mesenchymal stem cell, Extracellular vesicle, MicroRNA, Cell-free therapy

## Abstract

**Background:**

Periodontitis is a long-lasting inflammatory disorder of structures attached, which is significantly influenced by insufficient or dysregulated immune responses. Mesenchymal stem cells (MSCs) and their extracellular vesicles (EVs) are a promising therapeutic modality. This study explores the therapeutic efficacy of EVs derived from miR-146a-overexpressing bone marrow-derived mesenchymal stem cells (BMSCs) in a ligature-induced periodontitis model.

**Results:**

BMSCs were transfected to overexpress miR-146a, and their EVs were isolated. Periodontitis was induced in 30 female C57Bl/6 mice and divided into three groups: a control group receiving PBS, a miR-control EV-treated group, and a miR-146a-EV-treated group. EVs were administered subgingivally, and clinical parameters were evaluated. Levels of proinflammatory cytokines interleukin (IL)-1β, IL-8, tumor necrosis factor (TNF)-α, and IL-6, as well as anti-inflammatory cytokines IL-10, IL-4, and transforming growth factor (TGF)-β, were quantified using enzyme-linked immunosorbent assay (ELISA) and quantitative real-time polymerase chain reaction (PCR). Phosphorylated p38, JNK, ERK1/2, and NF-κB p65 were also quantified by ELISA, and TRAF6 and IRAK1 mRNA and protein levels were assessed to evaluate inflammatory signaling pathways. In the gingival tissue and systemic circulation, the proinflammatory cytokines IL-1β, IL-8, TNF-α, and IL-6 were significantly downregulated, while the levels of the anti-inflammatory mediators IL-10, IL-4, and TGF-β were significantly increased in the miR-146a-EV group when compared with the control group. miR-146a-EV treatment significantly reduced MAPK and NF-κB activation while downregulating TRAF6 and IRAK1 expression in gingival tissues.

**Conclusion:**

miR-146a-transduced MSC-derived EVs ameliorated ligature-induced periodontitis by modulating local and systemic cytokine expression. These results indicate that miR-146a-EVs can be a novel, cell-free therapy for the treatment of periodontitis.

## Background

Periodontitis is a heterogeneous, complex, chronic inflammatory disease often associated with the accumulation of dental plaque. This is characterized by progressive loss of the tooth support structures, including the periodontal ligament and alveolar bone [1]. Chronic hyperglycemia, poor oral hygiene, smoking, genetic predisposition, hormonal changes, and systemic diseases can all play a role in the development of periodontitis. Even with improved preventive measures, periodontitis continues to be a health concern in the world because of its high prevalence and potential systemic involvement [2]. Although intensive surgical interventions are employed, these approaches often fail to achieve complete regeneration of lost periodontal tissues or target underlying inflammatory processes [3]. Therefore, there is an urgent need for new therapeutic strategies that can modulate the immune response and promote tissue regeneration.

Mesenchymal stem cells (MSCs) are multipotent cells that can give rise to diverse mesenchymal cell lineages [4]. They also possess immunomodulatory properties that make them an attractive potential therapeutic option for various inflammatory diseases such as periodontitis. Previous studies have reported the potential therapeutic applications of MSCs for periodontitis [5]. It was previously thought that upon administration or injection, MSCs migrated to the site of injury, engrafted into the tissue, and differentiated into tissue-specific cell types. Now, however, this view of MSC function is undergoing a reevaluation. The current paradigm is changing towards another mechanism called paracrine effect, where MSCs release biologically active molecules, including extracellular vesicles (EVs) that exert regenerative activity in damaged tissues without necessarily differentiating into new cells [6]. EVs are released from the endosomal system and contain a cargo of parent cells-derived miRNA, mRNA, and proteins [7]. MSC-EVs have become a potential cell-free therapeutic application for periodontitis, as they have an immunoregulatory effect. These EVs modulate the inflammatory response through inhibition of pro-inflammatory cytokines (such as IL-1β, TNF-α) and activation of anti-inflammatory molecules (such as IL-10), thereby ameliorating alveolar bone loss and promoting periodontal regeneration [5, 8]. It has been demonstrated that MSC-derived EVs inhibit osteoclast activity and modulate macrophage polarization toward an anti-inflammatory phenotype in preclinical models of periodontitis, and they contribute to tissue regeneration partially through trafficking bioactive molecules to immune cells at the site of inflammation [9]. This immune regulation also confers EVs an exceptional capacity to treat chronic inflammatory diseases such as periodontitis, in which tissue destruction is promoted by dysregulated host responses [10].

miRNAs are small, fine noncoding RNAs around 19–24 nucleotides long that modulate the expression of these genes through base pairing and control around 30% of mammalian protein-coding genes [11]. MiR-146a is a crucial factor for decreasing the inflammatory response through the regulation of important molecules such as IL-1 receptor-associated kinase 1 (IRAK1), tumor necrosis factor receptor-associated factor 6 (TRAF6), and the NF-κB pathway. These molecules are necessary for the subsequent signaling of pro-inflammatory cytokines [12]. MicroRNA-146a-5p secreted by MSCs has been shown in animal studies to have anti-inflammatory effects and play a role in tissue regeneration [13]. In periodontitis, miR-146a is a critical player in immune responses and functions to downregulate the TLR/NF-κB signaling pathway by targeting several key signaling molecules, thus preventing excessive inflammation caused by periodontal pathogens such as Porphyromonas gingivalis [14]. It has been shown that the overexpression of miR-146a in immune cells inhibits cytokine production in lipopolysaccharide-induced models of gingival inflammation, which may contribute to the prevention of disease progression [15]. By encapsulating it in EVs, delivery to inflamed periodontal tissues is facilitated, and its therapeutic effect in regulating host immune response and resolution of chronic periodontitis is further enhanced [16].

Here, we investigated the therapeutic potential of the application of miR-146a-EVs originating from miR-146a-overexpressing bone marrow-derived mesenchymal stem cells (BMSCs) in ligature-induced periodontitis. Ligature-induced periodontitis is a widely established animal model that captures the chronic inflammatory mechanisms operated in human periodontal disease [17].

## Methods

### Isolation and characterization of BMSCs

The MSCs were derived from human bone marrow under the criteria of informed consent and according to the Declaration of Helsinki, from anonymous donors. The mononuclear cell separation from bone marrow was performed by density gradient centrifugation with Ficoll. These single cells were then plated into tissue culture plates at 2 million cells/ well. The cells were cultured (passages 3–11) in 10% human platelet lysate, 1% glutamine, and 1% penicillin-streptomycin-enriched MSC basal medium. Non-adherent cells were removed by changing the culture medium after 24 h incubation. Adherent cells were cultured until confluent and then passaged at a 1:3 ratio for further experimentation [18]. hBMSCs were chosen as the source of EVs owing to their strong immunomodulatory capacities, their low immunogenicity in xenogeneic systems, and their immediate translational implications for the treatment of human periodontitis.

To identify BMSCs, cells of passages 3–5 were subjected to extensive characterization. The expression of specific surface markers was tested through flow cytometry. CD73 and CD105 were identified as positive markers, while CD34 and CD45 were identified as negative markers. Their stem cell properties were further confirmed by evaluating their capacity to differentiate into various lineages. The cells were induced to differentiate into both osteogenic and adipogenic lineages. Calcium deposits produced during osteogenic differentiation were identified by Alizarin Red staining, and lipid droplets generated during adipogenic differentiation were observed by Oil Red O staining.

### BMSCs transfection with miR-146a

According to the manufacturer’s protocol, BMSCs were transfected in a 1:100 ratio of control mimic (mimic NC) or miR-146a mimic by Lipofectamine 3000 (Invitrogen, United States). The hsa-miR-146a-5p mirVana miRNA mimic (mature sequence: 5’-UGAGAACUGAAUUCCAUGGGUU-3’; catalog no. MC10722) and mirVana miRNA Mimic Negative Control #1 (mimic NC; a proprietary non-targeting scrambled sequence validated to not affect known miRNA functions) were obtained from Thermo Fisher Scientific (Invitrogen, United States). In brief, BMSCs were cultured in 80% confluence with Opti-MEM medium and incubated for 72 h with Lipofectamine 3000 reagent (Thermo Fisher Scientific) in the presence of miR-146a mimic or control mimic.

### EV isolation and characterization

When the BMSCs reached 85–90% confluency, EVs were collected from the BMSCs that were transfected with miR-146a mimic or control mimic. Before isolation of EVs, culture supernatants were replaced with EV-depleted fetal bovine serum (FBS) that had been exhaustively centrifuged at 4 °C (first at 500 × g for 20 min and subsequently at 18,000 × g for 30 min) and ultracentrifuged at 120,000 × g for 7 h. Before addition to the cell culture, the FBS containing the same batches of the EV-depleted was added into the cell culture, and filtered with a 0.22 μm membrane. Conditioned medium was collected at 48 h, after which it underwent sequential centrifugation: first for 20 min at 500 × g, then for 30 min at 20,000 × g. The resulting pellets from each spin were discarded, while the supernatant was retained. Subsequently, the supernatant was ultracentrifuged twice at 120,000 × g for 70 min. The pellet was resuspended in 1 mL of sterile PBS.

To evaluate the isolated EVs, the phenotypic marker CD63 was detected with flow cytometry (EV Isolation Kit CD63, Miltenyi Biotec, Germany), and protein levels were determined by the Protein Bicinchoninic Acid (BCA) Assay Kit (Solarbio, China). The size distribution and mean diameter of EVs were further analyzed by using a HORIBA SZ-100. For this analysis, samples were resuspended in PBS, kept at 25.2 °C, and analyzed at a 173° scattering angle using a 30% neutral density filter. The data were analyzed using the instrument’s software (Version 2.20). While additional characterization methods such as transmission electron microscopy and nanoparticle tracking analysis are recommended by MISEV2023 guidelines, they were not available during this study; therefore, we acknowledge this limitation and have based our EV identification on DLS size distribution and CD63 positivity, which are commonly accepted criteria in the literature.

### Ligature-induced periodontitis induction and treatment protocol

Female C57Bl/6 mice aged between 9 and 10 weeks were kept in a controlled room maintained at 24 °C with light/dark cycles under specific-pathogen-free conditions at the Yunnan University laboratory with access to sterile food and water. This study was reviewed and approved by the Institutional Animal Care and Use Committee of Yunnan University(Approval No.:YNU20261785), and all procedures were carried out following this institution’s guidelines. Female C57BL/6 mice were employed because they were more prone to ligature-induced periodontitis and bone loss than males, allowing for better visualization of anti-inflammatory therapeutic effects [19]. Periodontitis was induced based on the protocol described by Abe et al. [20]. In short, the mice were anesthetized by an intraperitoneal injection of a ketamine (100 mg/kg) and xylazine (10 mg/kg) mixture. The teeth in the mandible were ligated with a 5 − 0 silk suture. A 5 − 0 suture was navigated up the interdental space of the second and third molars with Dumont forceps and guided between the first and second molars. The 2nd molar was engaged with a loop of suture with tight stitching and was tied securely with a triple knot using suture-tying forceps. Spring scissors were used to trim excess suture material. The ligature was retained for 2 weeks for adequate development of chronic inflammation, substantial alveolar bone loss, and cytokine dysregulation, as described in standard protocols for C57BL/6 mice in which maximum pathogenesis is observed 7–14 days after ligature placement [5, 19].

The mice were monitored for two weeks, which was sufficient time for periodontitis to develop. Once periodontitis developed, mice were randomly assigned to three groups of 10 mice each using a computer-generated randomization schedule to ensure unbiased group assignment: a control group that received PBS, a group that was treated with miR-control EVs, and a group that was treated with miR-146a-EVs. Treatments were administered by subgingival injection (around the ligated tooth) at a dose of 50 µg total protein per mouse, given once at the end of the 2-week induction period to evaluate therapeutic effects on established disease. This timing and route target systemic and local anti-inflammatory modulation post-disease establishment [21]. To minimize bias, both the personnel administering the treatments and those performing outcome assessments were blinded to the group allocations throughout the experiment.

Animals were euthanized at the end of the experiment in accordance with ethical guidelines. Euthanasia was achieved by deep sedation using an anesthetic agent so that there would be no pain or distress to the animals during the process of euthanasia. Mice were first anesthetized by receiving an injection of a combination of ketamine (100 mg/kg) and xylazine (10 mg/kg) through the intraperitoneal injection, which was the same combination that was used to induce sedation for surgery. Once deep anesthesia was verified through the absence of pedal and corneal reflexes, animals were euthanized through cervical dislocation. This two-step procedure was utilized to adhere to institutional ethical standards and to follow recommended procedures for euthanasia in rodents, thereby permitting a rapid and humane death and allowing for the collection of blood and tissue samples for analysis thereafter.

### Cytokine assay

To evaluate the immunomodulatory effect of the treatments, an extensive analysis of the inflammatory microenvironment was done. Blood samples were taken at the end of the study period, both from treated and from control animals. Blood was allowed to clot at room temperature for 30 min, and serum was separated by centrifugation at 3,000 rpm for 10 min. The serum samples obtained were kept at -80 °C until assay analysis.

The levels of several pro-inflammatory and anti-inflammatory mediators were measured by enzyme-linked immunosorbent assay (ELISA), including interleukin (IL)-1β (R&D system, USA), IL-8 (MyBioSource, USA), tumor necrosis factor (TNF)-α (R&D system, USA), IL-6 (R&D system, USA), and anti-inflammatory cytokines IL-10 (R&D system, USA), IL-4 (R&D system, USA), and transforming growth factor (TGF)-β (R&D system, USA). All procedures followed the manufacturer’s instructions, and all samples were measured in triplicate to obtain more accurate results.

### Gene expression analysis in the gingival tissue

RNA was extracted from BMSC-EVs using TRIzol reagent, adhering to the manufacturer’s protocol. For quantification of miR-146a expression, RNA (1 µg) was subjected to reverse transcription to obtain cDNA using a MiRcute miRNA First-Strand cDNA Synthesis Kit. The 2^−ΔΔCt^ method was used to analyze the expression level relative to endogenous control on an ABI 7500 Real-Time PCR system. Amplification was performed using the MiRcute miRNA qPCR Detection Kit.

Gingival tissue was harvested from the ligated molar regions (maxillary/mandibular second molars) to assess local effects. Total RNA was isolated by RNeasy Mini Kit (Qiagen) following the manufacturer’s protocol. Briefly, the gingival tissue was homogenized in RLT buffer first, and then 70% ethanol was added to help RNA bind to the silica membrane inside the spin column. After removing contaminants through repeated washes, the RNA was eluted in RNase-free water and quantified with a NanoDrop spectrophotometer. For cytokine expression analysis, 1 µg of total RNA was reverse-transcribed to cDNA following the manufacturer’s instructions using High-Capacity cDNA Reverse Transcription Kit (Applied Biosystems). Cytokines, including TNF-α, IL-1β, IL-6, IL-8, IL-4, IL-10, and TGF-β, were detected by real-time PCR with specific primers. Reactions were performed at a total volume of 20 µL, including 10 µL SYBR Green Master Mix (Applied Biosystems), 1 µL cDNA template, and 0.5 µM of each primer. PCR amplification was carried out with an initial denaturation step of 10 min at 95 °C, followed by 40 cycles of 15 s at 95 °C, and 1 min at 60 °C for annealing and extension. Cytokine expression levels were adjusted through normalization to the housekeeping gene GAPDH, and the analysis was performed using the 2^−ΔΔCt^ method.

### Assessment of MAPK, NF-κB, TRAF6, and IRAK1 pathways activation by ELISA

To assess the activation status of major inflammatory signaling pathways, phosphorylated forms of p38 MAPK, JNK, ERK1/2, NF-κB p65, TRAF6, and IRAK1 were measured with commercially available ELISA kits (MBS3805594, MyBioSource, USA; DYC1387B-2, R&DSystem, USA; EMS2ERKP, Invitrogen, USA; MBS009329, MyBioSource, USA; MBS2882029, MyBioSource, USA; MBS457058, MyBioSource, USA; respectively). Gingival tissues were harvested and homogenized in ice-cold lysis buffer with protease and phosphatase inhibitors. Homogenates were centrifuged at 12,000 × g for 10 min at 4 °C, and the supernatants were used for analysis. The concentrations of p38 MAPK, JNK, ERK1/2, NF-κB p65, TRAF6, and IRAK1 were measured per the manufacturer’s protocols. The concentration of total protein in each gingival tissue lysate was quantified with the BCA assay before ELISA analysis. An equal amount of total protein was applied to each sample according to the manufacturer’s instructions. To eliminate variation in tissue weights and efficiency of protein extraction, levels of phosphorylated proteins were normalized to total protein content.

### Quantification of TRAF6 and IRAK1 gene expression by RT-PCR

To continue probing into the downstream regulators of the TLR/MyD88 signaling pathway, we measured mRNA levels of tumor necrosis factor receptor-associated factor 6 (TRAF6) and interleukin-1 receptor-associated kinase 1 (IRAK1) in gingival tissue. Total RNA was extracted with the RNeasy Mini Kit (Qiagen) following tissue homogenization. 1 µg of total RNA was subjected to reverse transcription into cDNA using the High-Capacity cDNA Reverse Transcription Kit. Quantitative RT-PCR was performed using SYBR Green Master Mix on the ABI 7500 Real-Time PCR System. Specific primers for TRAF6 and IRAK1 were used with GAPDH acting as the endogenous control. Relative expression levels were represented using the 2^−ΔΔCt^ method. All reactions were conducted in triplicate.

### Statistical analysis

IBM SPSS Statistics software version 26 was used for all statistical analyses. Data are presented as means ± standard deviations, and statistical significance was set to *p* < 0.05. Data were visualized using GraphPad Prism 8. Before performing statistical comparisons, the normality of data distribution was assessed using the Shapiro-Wilk test. In the case of datasets that followed a normal distribution, one-way analyses of variance (ANOVA) were used, using Tukey’s post hoc test for multiple comparisons. In this context, the Kruskal-Wallis test was used for non-parametric datasets.

## Results

### BMSCs characterization

**Fig. 1:**
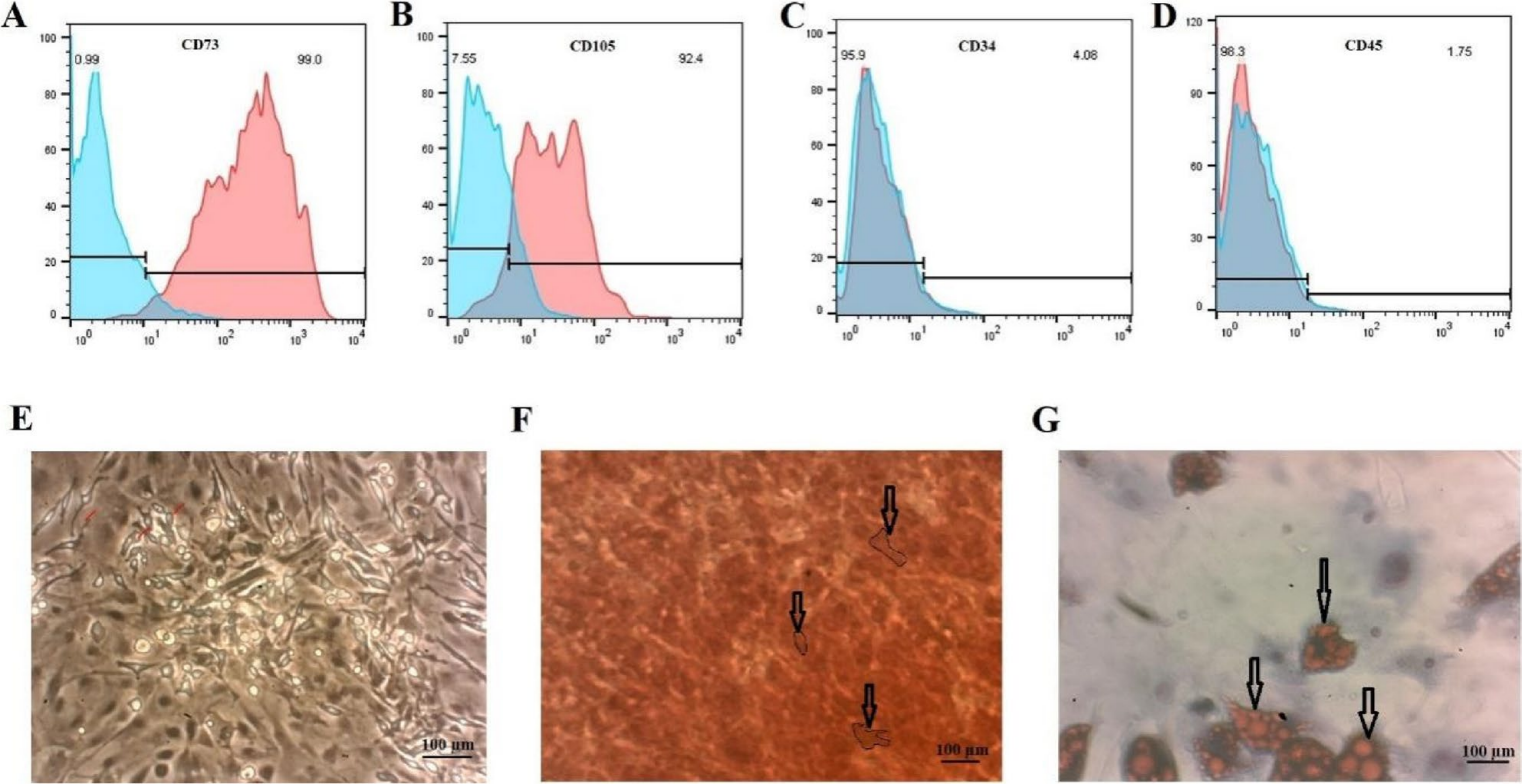
Characterization of BMSCs. **A**–**D** Flow cytometry analysis revealed positive expression of MSC markers CD73 (**A**) and CD105 (**B**), along with negative expression of hematopoietic markers CD34 (**C**) and CD45 (**D**). **E** BMSCs exhibited a spindle-shaped, fibroblast-like morphology with typical adherent growth. The red arrows show the kindle-shaped, fibroblast-like morphology. **F** Osteogenic differentiation was confirmed by Alizarin Red staining, which detected calcium mineralization (arrows indicate representative calcium nodules). **G** Adipogenic differentiation was demonstrated through Oil Red O staining, showing intracellular lipid accumulation (arrows indicate lipid droplets). BMSC; bone-marrow mesenchymal stem cell

In this study, we characterized BMSCs in great detail in terms of identity and stem cell properties. BMSCs were observed to possess the characteristics of MSCs by flow cytometry analysis of surface markers expressing CD34, CD45, CD73, and CD105, with positive results; CD73 and CD105 were positive, while CD34 and CD45 (Fig. 1A-D) were negative. This surface marker profile falls in line with the International Society for Cellular Therapy (ISCT) criteria for defining MSCs. BMSCs also exhibited a spindle-shaped, fibroblast-like morphology with typical adherent growth (Fig. 1E).To confirm their multipotency, the BMSCs were induced to differentiate into the osteogenic and adipogenic lineages. Calcium deposits, as visualized by Alizarin Red staining, confirmed osteogenic differentiation. Thus, the ability of BMSCs to form lipid droplets (confirmed by Oil Red O staining) confirmed that the cells could undergo adipogenic differentiation (Fig. 1F-G). Hence, these results indicated that the BMSCs maintained their multipotential differentiation capabilities and thus maintained their stem cell property.

### The BMSC-EVs Characterization

**Fig. 2:**
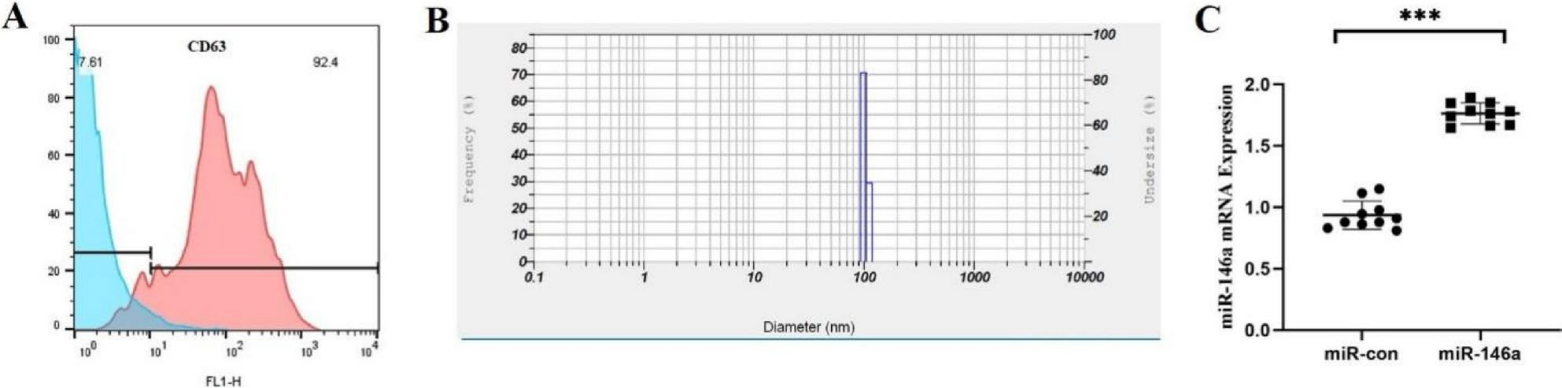
EVs isolation and characterization from BMSCs. **A** Flow cytometry analysis demonstrating high expression of exosomal marker (CD63) in isolated EVs, indicating their identity. **B** According to DLS analysis on a HORIBA SZ-100 instrument, BMSC-EVs had an average size of 102.7 nm. **C** qRT-PCR analysis showing that miR-146a in EVs derived from miR-146a-transfected BMSCs was significantly elevated compared with control EVs (mimic NC set to 1 as baseline endogenous level). Data shown as mean ± SD. (*** *p* < 0.001). Pink lines represent the expression of the respective markers, and blue lines represent the isotype control. DLS; dynamic light scattering, EV; extracellular vesicle, BMSC; bone-marrow mesenchymal stem cell

BMSC-EVs were successfully isolated and characterized to verify their identity and purity. EVs were isolated from the culture supernatants of BMSCs transfected with miR-146a mimic or control mimic. The isolated EVs showed a robust expression of well-known exosomal and small EV markers, CD63 (Fig. 2A). This confirmed that the isolated vesicles were EVs and did not contain any other cellular debris or non-EV particles. The above results indicated that the EVs isolated from BMSCs were of high purity and could be used for further functional studies. In addition, BMSC-EVs were analyzed to ensure the size and identity. Their average size was 102.7 nm as determined by DLS using a HORIBA SZ-100 instrument (Fig. 2B).

### miR-146a was effectively encapsulated within EVs

To assert the successful encapsulation of miR-146a into miR-146a-transfected BMSCs-derived EVs, quantitative RT-PCR analysis was performed. EVs were isolated from culture supernatants after transfecting BMSCs with a miR-146a mimic or a control mimic. MiR-146a expression levels were quantified by quantitative RT-PCR in the purified EVs. Expression levels were quantified using relative quantification (2^−ΔΔCt^ method), normalized to the mimic NC group (set to 1, representing baseline endogenous miR-146a levels without overexpression). Absolute quantification was not performed, as the focus was on fold-change relative to control.

The experiment showed that the expression levels of miR-146a in EVs from miR-146a-transfected BMSCs significantly increased in EVs isolated from miR-146a-transfected BMSCs compared to those from control cells (Fig. 2C). This result validates the efficient incorporation of miR-146a into the EVs during the transfection process.

### Cytokine assessment by ELISA

To assess how miR-146a-EVs altered the inflammatory microenvironment, serum samples were collected and analyzed for cytokine levels through ELISA. The levels of pro-inflammatory cytokines (IL-1β, IL-8, TNF-α, and IL-6) and anti-inflammatory cytokines (IL-10, IL-4, and TGF-β) were quantified.

Compared to the control (PBS-treated) and miR-control EV-treated group, downregulation of pro-inflammatory cytokines was significantly detected in the miR-146a-EV-treated group. In particular, the levels of IL-1β, IL-8, TNF-α, and IL-6 in the serum of animals treated with miR-146a-EVs were significantly decreased compared with those in control animals (except for IL-8 compared to miR-con EVs), indicating the inhibition of the inflammatory response.

**Fig. 3:**
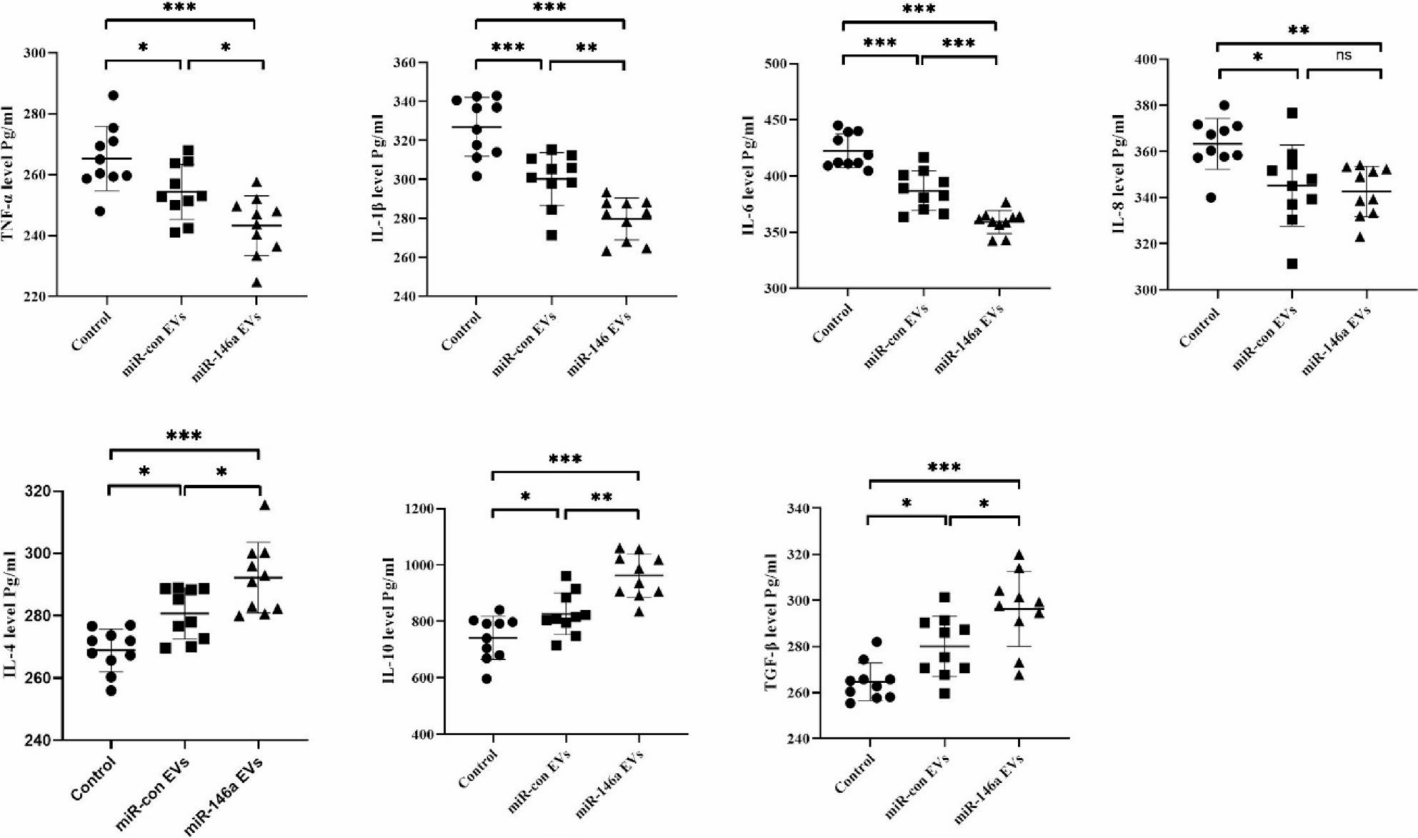
miR-146a-EVs-mediated systemic cytokine modulation. miR-146a-EVs treatment decreased pro-inflammatory cytokine levels and increased anti-inflammatory cytokine levels. Data shown as mean ± SD. *N* = 10/group. (*p < 0.05, ** *p* < 0.01, *** *p* < 0.001). IL; interleukin, TNF; tumor necrosis factor, TGF; transforming growth factor

Conversely, the anti-inflammatory cytokines (IL-10, IL-4, and TGF-β) were substantially higher in the miR-146a-EV-treated group than in the control and miR-control-EV-treated groups (Fig. 3). This elevation of inflammatory mediators also suggests a possible transition toward an anti-inflammatory microenvironment. These findings indicate that miR-146a-EVs positively influenced the cytokine balance within the systemic circulation, diminishing pro-inflammatory signals while simultaneously promoting anti-inflammatory responses.

### Cytokine evaluation by RT-PCR

To analyze the molecular mechanisms underlying the immunomodulation effects mediated by miR-146a-EVs, the levels of pro-inflammatory and anti-inflammatory cytokines in the gingival tissue were quantified using qRT-PCR. Cytokine mRNA levels of TNF-α, IL-1β, IL-6, IL-8, IL-4, IL-10, and TGF-β were evaluated and normalized to the housekeeping gene GAPDH.

Compared to both the control group (PBS-treated) and the miR-control EV-treated group, a significant downregulation of pro-inflammatory cytokine gene expression was found in the miR-146a-EV-treated group. The levels of TNF-α, IL-1β, IL-6, and IL-8 in the gingival tissue of animals treated with miR-146a-EVs were significantly lower compared with the expression in the gingival tissue of animals treated with PBS-treated and control-EVs. Importantly, this decrease demonstrates that miR-146a-EVs potently inhibited the local inflammatory response in the period.

**Fig. 4:**
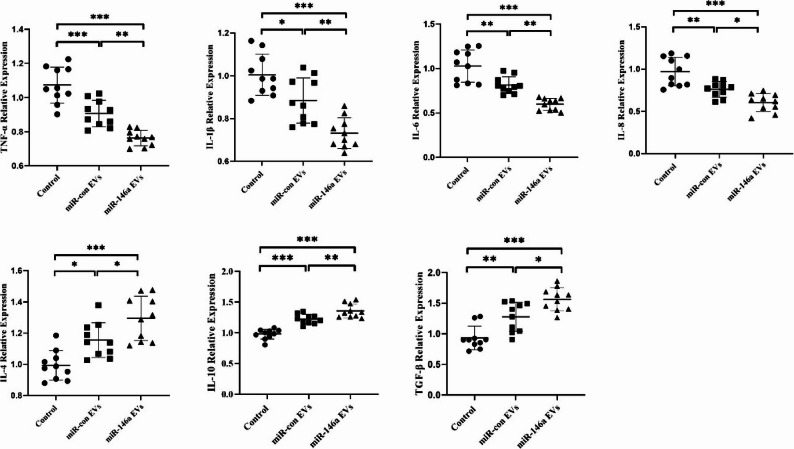
Local cytokine modulation in the gingiva by miR-146a-EVs. mRNA levels of pro-inflammatory (TNF-α, IL-1β, IL-6, IL-8) and anti-inflammatory (IL-4, IL-10, TGF-β) cytokines in gingival tissue as determined by quantitative RT-PCR. Mice treated with EVs overexpressing miR-146a showed significant inhibition of pro-inflammatory and upregulation of anti-inflammatory cytokines when compared with both PBS and miR-control EVs. Data shown as mean ± SD. *N* = 10/group. (*p < 0.05, ** *p* < 0.01, *** *p* < 0.001). IL; interleukin, TNF; tumor necrosis factor, TGF; transforming growth factor

Anti-inflammatory cytokines (including IL-4, IL-10, and TGF-β) were highly expressed in the miR-146a-EV treated group compared to either PBS control or miR-control EV groups (Fig. 4). The upregulation of anti-inflammatory gene expression in these monocytic cells indicates that miR-146a-EVs induced immunological polarization toward an anti-inflammatory microenvironment, which is required for tissue healing and regeneration of periodontitis.

### miR-146a-EV treatment suppresses MAPK/NF-κB pathway activation in the gingival tissue

**Fig. 5:**
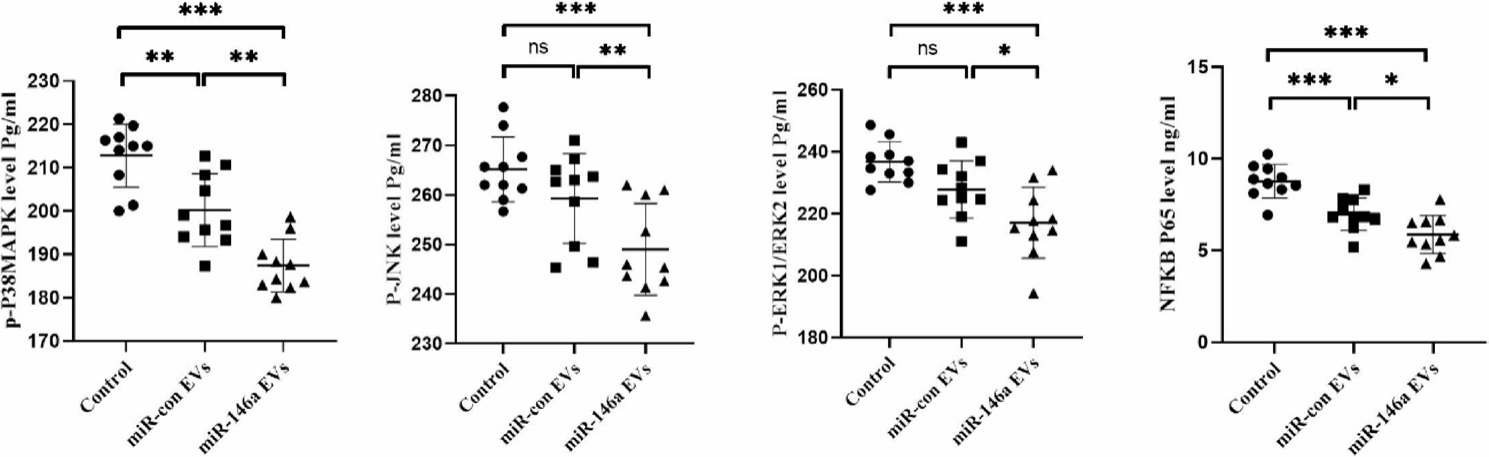
miR-146a-EV treatment inhibits MAPK and NF-κB activation in gingival tissue. ELISA was employed to measure levels of phosphorylated p38, JNK, ERK1/2, and NF-κB p65 in gingival tissue. The miR-146a-EV–treated group had significantly lower phosphorylation levels of all four inflammatory signaling molecules compared to the PBS group and miR-control EV group, indicating significant inhibition of intracellular inflammatory signaling pathways. Data are presented as mean ± SD (*N* = 10/group). (*p < 0.05, **p < 0.01, **p < 0.001). p-p38; phosphorylated p38 MAPK, p-JNK; phosphorylated c-Jun N-terminal kinase, p-ERK1/2; phosphorylated extracellular signal-regulated kinases 1/2, p-p65; phosphorylated NF-κB p65

To determine whether the anti-inflammatory effects of miR-146a-EVs correlate with inhibition of intracellular inflammatory signaling, the phosphorylated forms of key MAPK molecules (p38, JNK, ERK1/2), and phospho-NF-κB p65 were measured in gingiva tissues. Utilizing a combination of both PBS and miR-control EV groups, there were significant reductions in phospho-p38, phospho-JNK, phospho-ERK1/2, and phospho-NF-κB p65 levels in the miR-146a-EV group (Fig. 5). This level of suppression of MAPK activation and NF-κB is consistent with the reductions in pro-inflammatory cytokines; thus, we conclude that miR-146a-EVs blunt the intracellular inflammatory signaling cascade.

### miR-146a-EV treatment downregulates downstream TLR signaling molecules TRAF6 and IRAK1

**Fig. 6:**
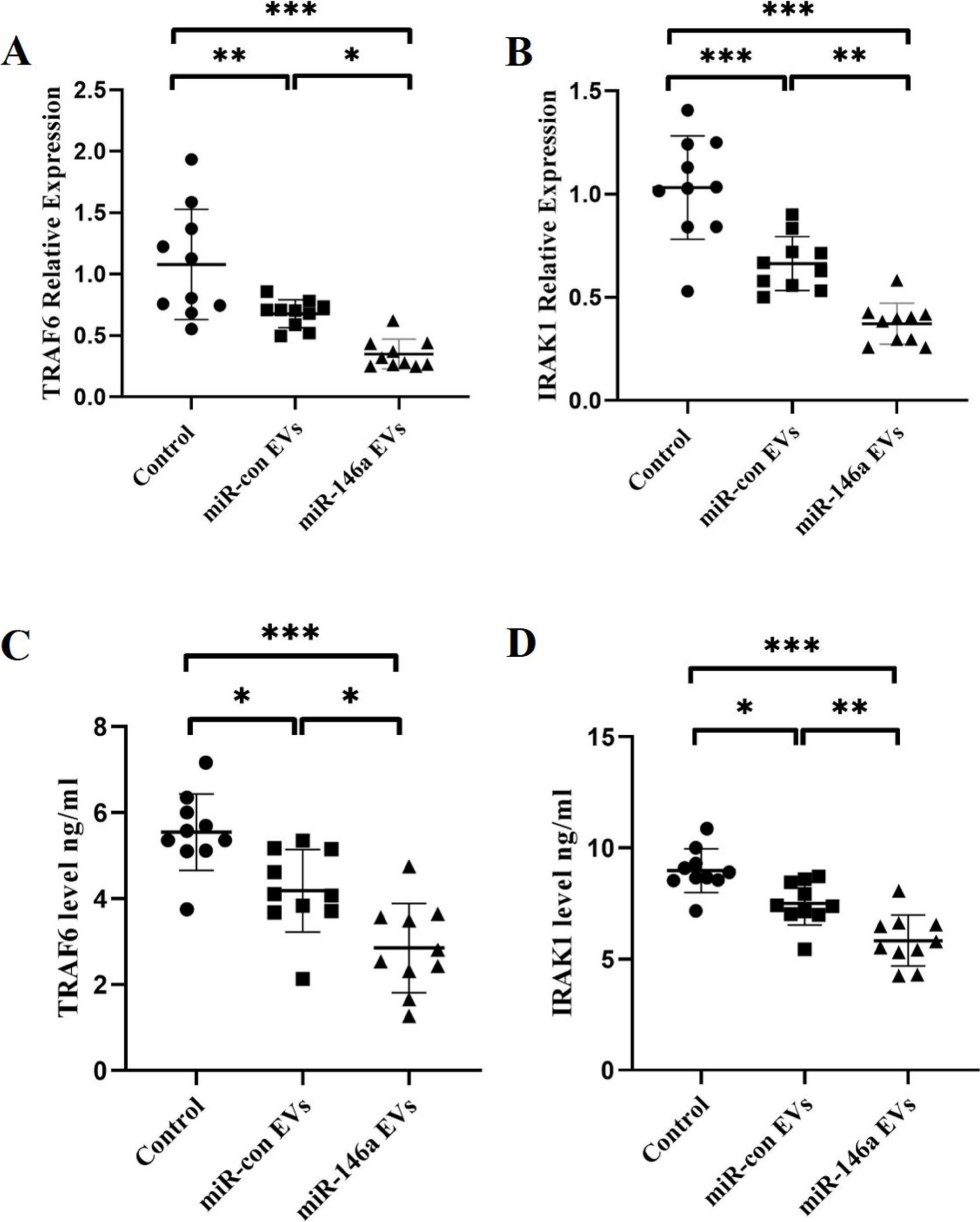
miR-146a-EV treatment downregulates TRAF6 and IRAK1 expression in the gingival tissue. **A**-**B** The relative mRNA expression levels of TRAF6 and IRAK1 were assessed through RT-PCR. **C**-**D** Protein levels of TRAF6 and IRAK1 in gingival tissue lysates were quantified by ELISA. The animals treated with the miR-146a-EV demonstrated a substantial decrease in both TRAF6 and IRAK1 mRNA and protein expression compared to both the PBS group and miR-control EV group, which is in agreement with known target genes regulated by miR-146a and inhibition of upstream TLR/MyD88 signaling. Data are presented as mean ± SD (*N* = 10/group). (*p < 0.05, **p < 0.01, **p < 0.001). TRAF6; tumor necrosis factor receptor-associated factor 6, IRAK1; interleukin-1 receptor-associated kinase 1

To further investigate whether miR-146a-EV treatment affected downstream components of the TLR/MyD88 pathway, TRAF6 and IRAK1 mRNA expression levels were determined in gingival tissues. Animals treated with miR-146a-EVs exhibited a significant reduction in TRAF6 and IRAK1 expression as compared to both control groups (Fig. 6A-B). Consistent with the mRNA findings, ELISA quantification of TRAF6 and IRAK1 protein levels in gingival tissue lysates revealed a marked decrease in the miR-146a-EV-treated group compared to PBS and miR-control EV groups (Fig. 6C-D). These results confirm that miR-146a-EVs effectively suppress both the transcription and translation of these key inflammatory adaptor molecules.

This downregulation aligns with established regulatory targets of miR-146a and, in part, supports the proposed mechanism by which miR-146a-containing EVs disrupt multiple key adaptor molecules, leading to activation of NF-κB and MAPK signaling. These findings suggest that miR-146a-EVs can perform their anti-inflammatory effects, at least in part, by suppressing TRAF6/IRAK1-dependent signaling.

## Discussion

The results demonstrated that miR-146a-EVs suppressed the expression of proinflammatory cytokines (IL-1β, IL-8, TNF-α, and IL-6) and enhanced the expression of anti-inflammatory factors (IL-10, IL-4, and TGF-β) at the local site and systemically, thus alleviating periodontitis. This correlated with diminished MAPK and NF-κB activation and reduced TRAF6/IRAK1 mRNA levels, known targets of miR-146a among the downstream TLR/MyD88 pathway adaptors, which is consistent with its role in downregulating the excessive inflammation [12, 22]. These results are consistent with prior studies demonstrating that MSC-EVs have anti-inflammatory effects in periodontitis by delivery of miRNAs [8, 9]. Transfected miR-146a-EVs also suppress NF-κB/MAPK signaling and TRAF6/IRAK1 expression in other inflammatory models [23]. Compared to non-engineered EVs, overexpression of miR-146a further improved the modulation of cytokines and the inhibition of the pathways, which support the further therapeutic amplification of miR-146a in periodontal diseases.

EV uptake is mainly mediated by endocytosis (clathrin- or caveolin-dependent), macropinocytosis, direct membrane fusion or other mechanisms, resulting in intracellular miR-146a cargo release in the recipient cells [24]. Subsequently, miR-146a is loaded onto the RNA-induced silencing complex (RISC) following internalization to target mRNAs, including IRAK1 and TRAF6, resulting in suppression of downstream NF-κB and MAPK signaling [22, 25]. This efficient delivery contributes to the local and systemic anti-inflammatory effects observed in our model.

Periodontitis manifests with a dysregulated host immune response to microbial biofilms that results in excessive inflammation and tissue destruction [26]. Traditionally, the management of periodontal disease has focused on mechanical debridement, surgical therapy, and antibiotics, but these methods alone rarely succeed in regenerating lost periodontal tissues in their entirety [27]. However, therapies based on stem cells, especially MSC-derived EVs, represent an attractive cell-free approach that retains the paracrine regenerative and immunomodulatory effects of MSCs, while avoiding the risks of direct stem cell transplantation [28]. Current data from this study also confirms this hypothesis, demonstrating that miR-146a-EVs modulate cytokine profiles and most probably also lead to the resolution of inflammation, which is an essential step in periodontal regeneration.

Interestingly, the miR-146a-EV-treated group showed a significant downregulation of pro-inflammatory cytokines (IL-1β, IL-8, TNF-α, and IL-6), indicating that miR-146a possesses a remarkably anti-inflammatory property in periodontitis. These cytokines are crucial in the pathogenesis of periodontal disease, as they magnify the inflammatory cascade, recruit immune cells, and mediate tissue destruction [29]. TNF-α and IL-1β are two important osteoclastogenesis mediators that have shown predominant alveolar bone resorption effects [30]. Downregulation of these cytokines suggests that miR-146a-EVs are counteracting inflammatory signaling pathways, possibly through inhibition of the proinflammatory NF-κB pathway, a major regulator of cytokine expression. Although miR-146a-EV treatment dramatically suppressed the IL-8 expression in the gingival tissue, no decrease in serum IL-8 level was detected. This apparent discrepancy is probably due to the mainly local effect of IL-8 in periodontal inflammation. IL-8 is produced primarily by resident gingival cells and infiltrating leukocytes at the site of infection, where it serves as a pivotal chemokine for the recruitment of neutrophils [31]. Hence, the local inhibition of IL-8 expression does not necessarily result in detectable effects in the systemic circulation, where IL-8 is produced by many other tissues and is rapidly cleared and diluted.

Moreover, the marked increase of several anti-inflammatory cytokines, especially IL-10 and TGF-β, indicates a transition to an anti-inflammatory phenotype, which is essential for tissue repair and homeostasis [32]. IL-10 belongs to a family of immunoregulatory cytokines that inhibit excessive inflammatory responses, namely, TGF-β, which is vital in mediating tissue regeneration by promoting deposition of extracellular matrix components and stimulation of fibroblast proliferation [33]. Higher levels of these cytokines in response to miR-146a-EV treatment suggest promising mechanisms for periodontal tissue restoration, complementing existing regenerative therapies.

Not only did miR-146a-EVs modulate systemic and local cytokine profiles, but they also had a substantial impact on intracellular signaling pathways responsible for the propagation of periodontal inflammation. In gingival tissue, treatment with miR-146a-EVs reduced the phosphorylation of p38, JNK, ERK1/2, and NF-κB p65 dramatically, suggesting effective suppression of the activation of the MAPK and NF-κB pathways. In addition, the gene expression of TRAF6 and IRAK1, two key upstream adaptor molecules and direct targets of miR-146a, was also significantly downregulated after miR-146a-EV treatment. These findings are consistent with other published data that elucidate miR-146a functions in immune regulation by targeting essential inflammatory pathways such as IRAK1 and TRAF6, which play a major role in NF-κB-mediated cytokine signaling [12, 34]. While miRNAs have been shown to play a role in translation and mRNA stability, miR-146a has been repeatedly described to decrease the expression of TRAF6 and IRAK1 at the mRNA level in inflammation-related contexts [23]. Here, we demonstrated that the decreased levels of TRAF6 and IRAK1 transcripts were paralleled by a profound inhibition of MAPK and NF-κB activation, thus functionally supporting that the miR-146a/TRAF6/IRAK1 signaling pathway was targeted in vivo. In summary, the results of these studies indicate that miR-146a-EVs prevented inflammation systemically and locally by directly inhibiting the signaling pathways that are responsible for upregulating inflammation both on a cellular and gene expression level. This mechanistic evidence supports the efficacy of miR-146a-EVs as a cell-free treatment approach to limit periodontal inflammation through the regulation of both upstream and downstream signaling pathways of inflammation. The suppression of MAPK/NF-κB activation and TRAF6/IRAK1 expression is consistent with miR-146a’s role in negative regulation of TLR signaling. Functional validation (e.g., using NF-κB inhibitors like BAY 11-7082, p38 inhibitor SB203580, or miR-146a mimic/inhibitor rescue assays in vitro) could further confirm causality.

This study allows the use of the ligature-induced periodontitis model, which is a well-accepted platform on which to build a model of chronic inflammation that is similar to chronic inflammation in human periodontitis. This model effectively recapitulates the pathological features of disease, including alveolar bone loss and gingival inflammation, providing a platform to evaluate therapeutic interventions in a reproducible manner [35]; however, alongside the insights into disease posed by this animal model, it must also be contextualized for its restrictions. Nevertheless, clinical studies should be performed to validate the effect of miR-146a-EVs in human periodontitis, since the immune response of rodents and humans differs.

While the immunomodulatory function of miR-146a is well-defined (i.e., negatively feeds back on NF-κB through targeting IRAK1/TRAF6) [22], miR-146a-EVs present unique benefits when considering the severity of other miRNA-based or immunomodulatory approaches to periodontitis. Instead of direct miRNA mimics/inhibitors or artificial nanoparticles, miR-146a-EVs provide natural encapsulation and protection of miR-146a from degradation by nucleases, improved stability, and enhanced cellular absorption via intrinsic pathways (for example, endocytosis, membrane fusion), resulting in functional intracellular delivery to target cells, including macrophages and gingival fibroblasts [10, 24]. In contrast to whole MSC transplantation, which is associated with immune rejection, tumorigenesis, or low engraftment, miR-146a-EVs are cell-free, have low immunogenicity, and maintain strong paracrine immunomodulation with fewer safety issues [10, 36]. When compared with unmodified MSC-EVs or other miRNA cargos (e.g., miR-155 or miR-21), miR-146a overexpression intensifies targeted inhibition of TLR/NF-κB/MAPK pathways, causing a stronger decrease of pro-inflammatory cytokines (IL-1β, TNF-α, IL-6) and enhancement of anti-inflammatory mediators (IL-10, TGF-β), as demonstrated in this and other inflammation models. These attributes make miR-146a-EVs an excellent cell-free candidate to finely tune chronic periodontal inflammation with added potential for enhancing tissue regeneration [37, 38].

Though these findings are promising, many challenges remain. Scalability and reproducibility of EV generation are essential for successful clinical translation. Protocols for EV isolation, characterization, and dosing should be standardized to ensure reproducible therapeutic effects. While ELISA was used to quantify TRAF6 and IRAK1 protein levels due to limited gingival tissue, Western blotting would provide additional validation. Future studies using in vitro models or larger samples are encouraged to further confirm these findings through complementary protein analyses. Another important limitation is that we did not directly measure alveolar bone loss (e.g., by using micro-CT to measure the CEJ-ABC distance or by histological assessment of bone height/osteoclast activity), but mainly analyzed early inflammatory cytokine profiles and signaling pathway modulation. Although the decreases in pro-inflammatory cytokines and MAPK/NF-κB activation observed in this study are indicative of potential protection from tissue destruction, a functional demonstration for the reduced destruction of periodontal tissues is required. Future studies should aim to incorporate micro-CT or stereomicroscope-based linear quantification of alveolar bone loss at multiple sites surrounding ligated molars in order to confirm biological significance. Finally, the lack of direct in vivo monitoring of EV biodistribution, retention, and clearance following subgingival administration. Local administration is based on targeted retention in the periodontal pocket, but lymphatic drainage or phagocytosis can negatively affect long-term exposure in inflamed gingival tissue. Future studies with labeled EVs (e.g., DiI/PKH26 fluorescence or luciferase-tagged) should employ imaging (IVIS) or histology to verify local retention and to relate pharmacokinetics to therapeutic effects.

## Conclusion

In summary, this study proposes a new mechanism of action whereby miR-146a-EVs from MSCs have a dual therapeutic role and regulate systemic and bone inflammation in periodontitis. miR-146a-EVs were able to promote a microenvironment suitable for tissue healing, through the downregulation of pro-inflammatory cytokines and the upregulation of anti-inflammatory cytokines. These data strongly advocate that miR-146a-EV could be used as a new cell-free therapy for periodontitis treatment. In the future, long-term bone regeneration needs to be assessed, localized EV delivery (e.g., gels or scaffolds) should be tested, changes at the protein level have to be confirmed, and results should be validated in a large animal model or human trials. EV production and dose must be standardized, which will be essential in the clinical translation. Overall, miR-146a-EVs could be a potential cell-free therapeutic option for the regulation of inflammation in periodontitis.

## Data Availability

The data that support the findings of this study are available from the corresponding author, D. Huang, on special request.

## Abbreviations

ANOVA: Analysis of Variance
ARRIVE: Animal Research: Reporting of In Vivo Experiments
BCA: Bicinchoninic Acid
BMSC: Bone Marrow-Derived Mesenchymal Stem Cell
cDNA: Complementary DNA
ELISA: Enzyme-Linked Immunosorbent Assay
EV: Extracellular Vesicle
FBS: Fetal Bovine Serum
GAPDH: Glyceraldehyde 3-Phosphate Dehydrogenase
IL: Interleukin
IRAK1: Interleukin-1 Receptor-Associated Kinase 1
ISCT: International Society for Cellular Therapy
miR-146a / miRNA: microRNA-146a / microRNA
MSC: Mesenchymal Stem Cell
NF-κB: Nuclear Factor Kappa-Light-Chain-Enhancer of Activated B Cells
PBS: Phosphate-Buffered Saline
PCR: Polymerase Chain Reaction
qRT-PCR / RT-PCR: Quantitative Real-Time Polymerase Chain Reaction / Reverse Transcription Polymerase Chain Reaction
SD: Standard Deviation
TGF-β: Transforming Growth Factor Beta
TNF-α: Tumor Necrosis Factor Alpha
TRAF6: Tumor Necrosis Factor Receptor-Associated Factor 6
